# Hemorrhage associated with hepatic artery pseudoaneurysms after regional chemotherapy with floxuridine: case report

**DOI:** 10.1186/1477-7800-5-17

**Published:** 2008-07-11

**Authors:** Panagiotis Samaras, Thomas Pfammatter, Bernhard C Pestalozzi

**Affiliations:** 1 Department of Oncology, University Hospital Zurich, Zurich, Switzerland; 2 Institute of Diagnostic Radiology, University Hospital Zurich, Zurich, Switzerland

## Abstract

Pseudoaneurysms of the hepatic artery are a rare complication in patients with primary or secondary liver tumors treated with intra-arterial chemotherapy. We present two patients who developed this complication after placement of a catheter system into the gastroduodenal artery and initiation of regional chemotherapy with floxuridine. Diagnosis was made after symptomatic bleeding occurred, necessitating emergency angiography with coil embolization. Pseudoaneurysms usually occur after mechanical damage of the vessel wall, but the chemical toxicity of floxuridine may add to the development of vascular impairment.

## Background

Hepatic artery chemotherapy (HAC) was developed in the early 1960s to treat large, unresectable primary and secondary liver tumors [1,2]. This approach is based on the rationale that local therapy through the hepatic artery increases the exposure of the liver tumors to cytotoxic agents, leading to higher response rates while limiting systemic side effects like myelosuppression, mucositis and diarrhea. Surgical or percutaneous placement of a subcutaneous device allows chemotherapy to be delivered continuously into the gastroduodenal artery via a catheter. Regional chemotherapy predominantly for the treatment of colorectal liver metastases was increasingly used in the late 1980s and 1990s because of the limited efficacy of available systemic treatment options [3-9]. This approach was also evaluated in the treatment of unresectable primary liver tumors and as adjuvant treatment after metastasectomy [10-14]. However, the application of floxuridine-deoxy-ribose (FUDR), a prodrug of 5-fluorouracil (5-FU) with a high liver extraction fraction, into the hepatic artery can cause several complications. Biliary toxicity is the dose limiting side effect of HAC with FUDR, usually indicated by an increase of bilirubin and liver enzymes [15-18]. Other infrequent complications are gastric and duodenal ulcerations, mainly due to misperfusion. Rarely thrombosis, nausea, vomiting and diarrhea occur [16,19,20]. Pocket infections, development of cholangitis and liver abscesses have also been reported [21].

A rare complication of HAC is the formation of pseudoaneurysms of the hepatic artery. In this article we describe two patients who developed aneurysmatic cavities of the hepatic artery after regional chemotherapy with FUDR and presented with hemorrhage.

## Case presentation

### Case 1

A 62-year-old male patient presented in May 2001 with initially unresectable metachronous liver metastases of a surgically treated rectal carcinoma. Cholecystectomy and implantation of an infusion system (Arrow pump, Codman 3000, Johnson & Johnson, Raynham, Mass.) were carried out by laparotomy. From June 2001 to October 2001 he received four cycles of neoadjuvant HAC with FUDR monotherapy in a total dose of 1100 mg. In November 2001 hemihepatectomy of the right lobe could be performed after downsizing of the metastases. Five months later, he developed a solitary liver metastasis. In April 2002 a wedge-resection was performed, followed by one cycle of adjuvant HAC with FUDR in May 2002. 15 days after restart of regional therapy the patient presented with abdominal pain. Computed tomography revealed a large retroperitoneal hematoma and a ruptured pseudoaneurysm of the hepatic artery at the site of the catheter tip.

**Figure 1:**
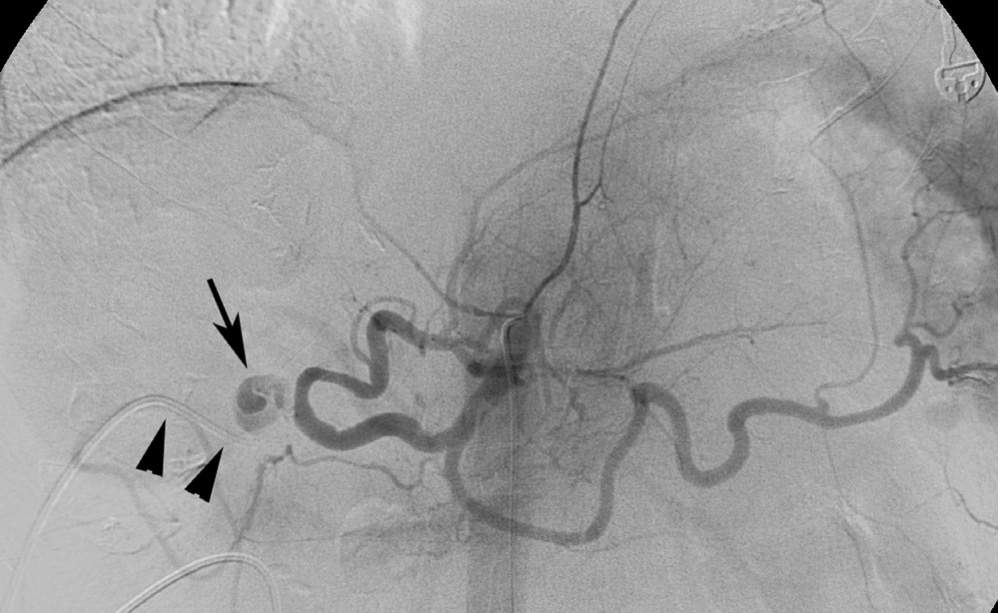
**Celiac angiogram**. The celiac angiogram shows a pseudoaneurysm *(arrow) *of the proper hepatic artery located next to the origin of the gastroduodenal artery, which is occluded by the chemotherapy infusion catheter *(arrowheads)*. The right hepatic artery is missing after hemihepatectomy.

**Figure 2:**
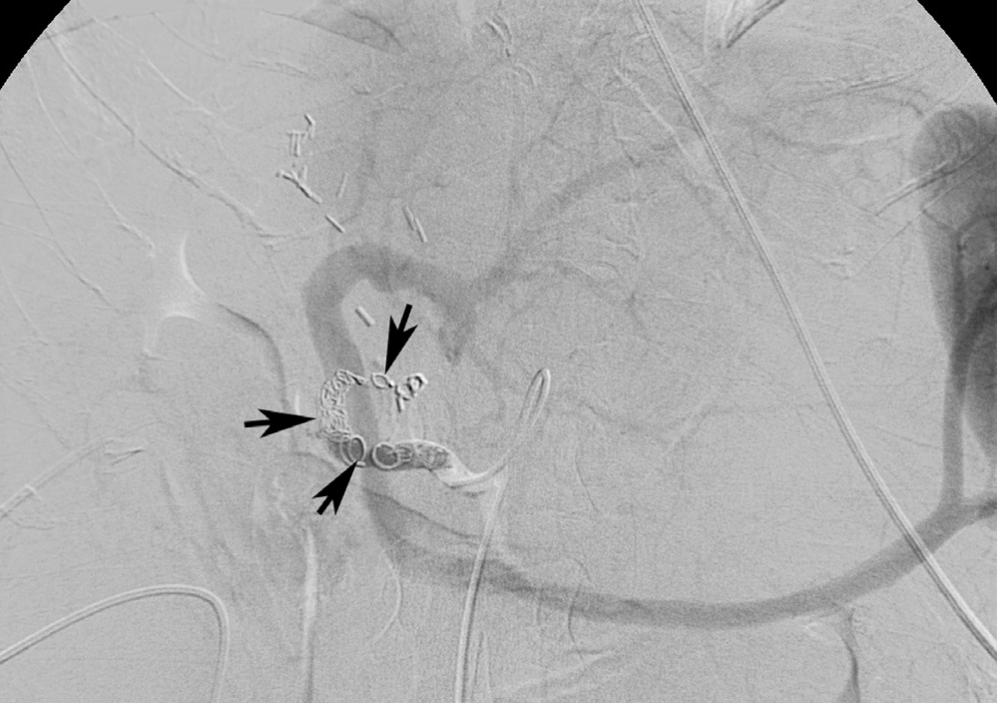
**Completion celiac angiogram**. In the completion celiac angiogram the common hepatic artery is occluded by the microcoils *(arrows)*. The liver is supplied uniquely by the patent portal vein.

In a subsequent angiography via a transfemoral access a stent-graft was placed into the hepatic artery in order to preserve arterial hepatic perfusion. This led only to a partial exclusion of the aneurysm and was complicated by a dissection of the hepatic artery, reflecting its friability. Definitive treatment was finally achieved by coil embolization (Figures 1, 2). After clinical stabilization, the pump system was removed. Regional therapy was discontinued and the patient recovered uneventfully.

### Case 2

**Figure 3:**
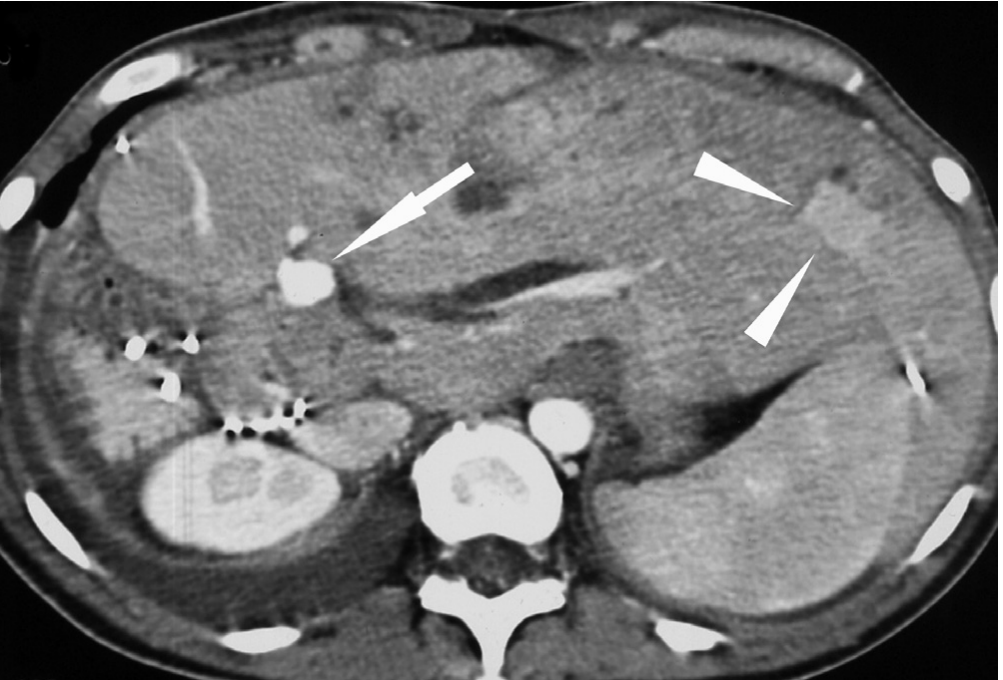
**Contrast-enhanced abdominal CT**. The left liver lobe is hypertrophied after extended right hemihepatectomy. There is an arterial pseudoaneurysm *(arrow) *close to the hepato-enteric anastomosis. A hypervascular metastasis is depicted on the same cut *(arrowheads)*.

A 38-year-old female patient was diagnosed with a multifocal hepatocellular carcinoma in July 2000. Hemihepatectomy of the right lobe, resection of segment I and cryosurgery of a satellite lesion in segment II were performed one month later. Because of recurrence of the tumor, a wedge resection of segment III/IV followed in February 2001. Concurrently, an Arrow pump system was implanted in a subcutaneous pocket on the lower right side of the abdomen. The catheter tip was placed at the origin of the gastroduodenal artery. From May 2001 to September 2001 she received HAC with 5 cycles of FUDR in a total dose of 600 mg to slow down tumor growth until orthotopic liver transplantation could be performed. In October 2001 the treatment had to be discontinued because of biliary toxicity with elevated liver enzymes and bilirubin. Five months later she had progressive disease and HAC was started again. Because of the previous toxicity, FUDR was administered in reduced dose. After completion of the fourth cycle, she presented to our emergency room with jaundice, fever and epigastric pain in June 2002. She was hospitalized and antibiotic treatment with ciprofloxacin was started. After isolation of *Klebsiella pneumoniae *from the blood culture, antibiotic treatment was changed to piperacillin and tazobactam. Percutaneous transhepatic cholangiography showed multiple liver abscesses. A few days later the patient developed severe hematemesis and melena necessitating blood transfusions. Gastroscopy showed blood remnants without any lesions of the gastrointestinal mucosa and hemobilia was seen in a repeated cholangiogram. Finally, computed tomography revealed a pseudoaneurysm of a segmental left hepatic artery, about 5 cm from the site of the catheter tip close to the hepato-enteric anastomosis (Figure 3). The development of a hepatic artery-biliary fistula was assumed to be the cause of the gastrointestinal bleeding.

**Figure 4:**
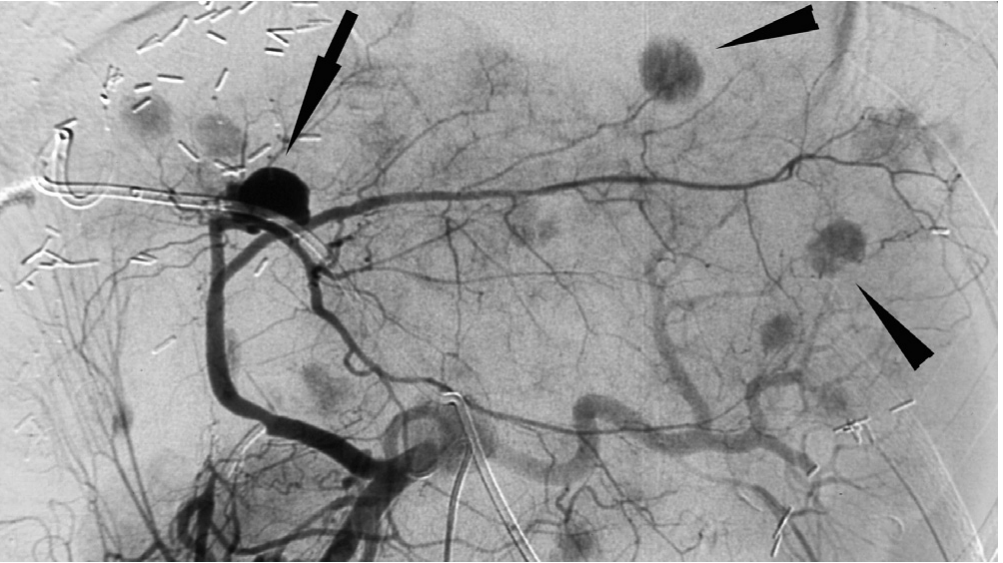
**Celiac angiogram**. Celiac angiogram with pseudoaneurysm of a left segmental artery *(arrow) *and disseminated metastases *(arrowheads)*.

**Figure 5:**
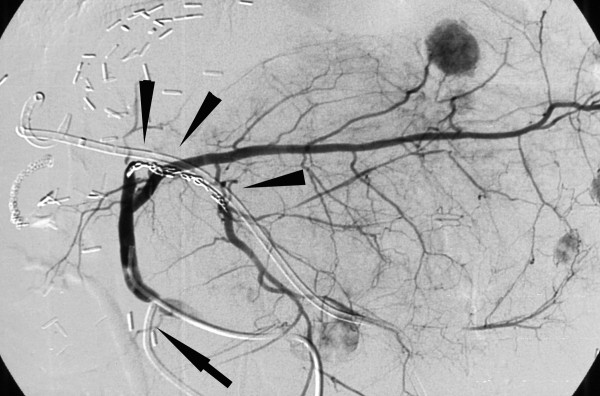
**Completion hepatic arteriogram**. Coils in the segmental artery III *(arrowheads) *after embolisation. Due to hepatic redistribution the embolized segmental artery is opacified distally to the coils. A hepatic chemotherapy infusion catheter had been inserted into the gastroduodenal artery *(arrow)*.

Angiography was performed via a transfemoral access, and after placement of a 4 French catheter in the segmental artery the aneurysm was occluded by coil embolization (Figures 4, 5). A few days later, the fever and jaundice resolved completely. The arrow pump was removed and no further chemotherapy was applied. The patient recovered uneventfully.

## Discussion

Pseudoaneurysms of the hepatic artery due to placement of a catheter into the gastroduodenal artery and treatment with continuous regional chemotherapy are described infrequently in the literature [9,13,22]. They can remain clinically silent even after continuation of the treatment [23]. A few case reports describe the development of hemorrhage after rupture of the pseudoaneurysm or fistulation to a bile duct or the intestine [24-26]. Pseudoaneurysms usually develop after percutaneous or surgical procedures as a result of dissection of the vessel. A penetrating trauma leads to a disruption of the media without blood seepage through the adventitia. The late development after multiple courses of intra-arterial chemotherapy, as described in our two patients, emphasises the role of HAC as a potential cause. Hepatic artery aneurysms account for 20% of all visceral aneurysms [27]. The rate of rupture was reported to be as high as 65%, with a mortality rate of 20% [28]. Symptoms of rupture include abdominal pain in case of retroperitoneal bleeding and hematemesis or melena, if the aneurysm is in contact with the biliary tract or the duodenal lumen. Early interventional therapy is necessary to control hemorrhage. The preferred approach is a hepatic angiography with subsequent coil embolization because pseudoaneurysms are usually located adjacent to the hilum of the liver, making surgery difficult [29-33]. Major risks of this procedure include arterial dissection, artery occlusion, thromboembolic phenomena and intraprocedural rupture of the aneurysm. Recently, promising results were reported with endovascular stent-graft placement, but long-term results are still lacking [34,35]. There are also reports of successful treatment with computed tomography guided percutaneous embolization and ultrasound guided percutaneous thrombin injection [36,37].

Considering the development and widespread use of more effective systemic chemotherapy, the use of regional chemotherapy decreased in the last years. A recent meta-analysis showed that FUDR-based HAC alone does not improve response rates and outcome compared with 5-FU based combination chemotherapy [38]. However, the role of regional chemotherapy with FUDR in primary liver tumors and in combination with efficient systemic chemotherapy in colorectal liver metastases has not been defined yet. HAC should be utilized only in high-volume centers with expertise in this field due to the high technical demands of this procedure, since the rate of complications is clearly related to the surgeon's experience [39]. Clinicians should be aware of the possibly life threatening complications in patients treated with HAC. Because of the unpredictable course of pseudoaneurysms, early treatment with interventional radiological techniques should be considered. In the case of our two patients, angiography with coil embolization was necessary to control the critical condition.

## Conclusion

Hepatic artery pseudoaneurysms may complicate regional chemotherapy with floxuridine. Prompt angiography with coil embolization is the preferred treatment to avoid life threatening hemorrhage in these patients.

## Consent

Both patients gave written informed consent for publication of their data for scientific purposes.

## Competing interests

The authors declare that they have no competing interests.

## Authors' contributions

PS and BCP collected the data and wrote the article. TP provided the angiography pictures, contributed to the analysis and interpretation of the data and revised the manuscript. All authors read and approved the final manuscript.
